# An Equivalent Consumption Minimization Strategy for a Parallel Plug-In Hybrid Electric Vehicle Based on an Environmental Perceiver

**DOI:** 10.3390/s22249621

**Published:** 2022-12-08

**Authors:** Shilin Pu, Liang Chu, Jincheng Hu, Shibo Li, Zhuoran Hou

**Affiliations:** 1 College of Automotive Engineering, Jilin University, Changchun 130022, China; 2 Department of Aeronautical and Automotive Engineering, Loughborough University, Loughborough LE11 3TU, UK

**Keywords:** plug-in hybrid electric vehicle (PHEV), energy management strategy (EMS), equivalent consumption minimization strategy (ECMS), real-time traffic state perception, deep learning

## Abstract

An energy management strategy is a key technology used to exploit the energy-saving potential of a plug-in hybrid electric vehicle. This paper proposes the environmental perceiver-based equivalent consumption minimization strategy (EP-ECMS) for parallel plug-in hybrid vehicles. In this method, the traffic characteristic information obtained from the intelligent traffic system is used to guide the adjustment of the equivalence factor, improving the environmental adaptiveness of the equivalent consumption minimization strategy (ECMS). Two main works have been completed. First, a high-accuracy environmental perceiver was developed based on a graph convolutional network (GCN) and attention mechanism to complete the traffic state recognition of all graph regions based on historical information. Moreover, it provides the grade of the corresponding region where the vehicle is located (for the ECMS). Secondly, in the offline process, the search for the optimal equivalent factor is completed by using the Harris hawk optimization algorithm based on the representative working conditions under various grades. Based on the identified traffic grades in the online process, the optimized equivalence factor tables are checked for energy management control. The simulation results show that the improved EP-ECMS can achieve 7.25% energy consumption optimization compared with the traditional ECMS.

## 1. Introduction

With the growth of a vehicle’s preserved volume, large amounts of greenhouse gas (GHG) emissions, and massive consumption of petroleum resources, the problems of global warming and the energy crisis have become severe [1,2,3]. Developing environmentally friendly and energy-efficient transportation is critical to mitigating these problems. Compared with hybrid electric vehicles (HEVs), conventional internal combustion engine vehicles (ICEVs), battery electric vehicles(BEVs) and hydrogen fuel cell vehicles (HFCVs), have their own advantages and disadvantages [4]. Although HFCVs emit water and heat, which are harmless greenhouse gases [5] and are the most environmentally friendly, the cost of hydrogen fuel infrastructures will limit the applications. ICEVs are limited by the thermal efficiencies of engines, resulting in excessive NOx emissions. Although BEVs have the advantages of zero emissions and high electrical energy utilization, if the source of the electric energy is calculated from a coal-burning plant, the energy conversion efficiency of this process will not be high because the heat energy generated by coal and natural gas is converted into electric energy, and greenhouse gases cannot be completely avoided in the process [6]. As a bridge connecting ICEV and BEV, HEVs can maximize the energy conversion efficiency at the vehicle end through the management of multiple energy sources. It is a practical solution for the commercialization to reduce emissions and has been paid close attention to among scholars and practitioners [7]. As an improved form of HEV, the plug-in hybrid electric vehicle (PHEV) has massive advantages in energy-saving performances, benefiting from large capacities and improved engines [8,9]. However, the essential solution to boost the potential of PHEV with a high control of freedom is an effective energy management strategy (EMS), which is a considerable task that needs to be solved.

At present, the massive amounts of EMSs utilizing single-vehicle data are being explored, and some are applied in industrial circles. General EMSs are classified into rule-based EMSs, global optimization-based EMSs, and instantaneous optimization-based EMSs. Among them, EMSs based on rules develop rules for controlling the working conditions of the engine and motor, so that the reference values expressed as the vehicle’s required power or the state of charge (SOC) of the battery are approached. Benefiting from the low complexity of the calculation, the methods are applied to the developers of production vehicles, who are divided into the algorithm based on deterministic rules and fuzzy logic [10,11]. On the flip side, the kind of strategy that relies on expert experience requires time, which leads to energy consumption reaching the global optimum in a tough manner. Due to the intelligent group optimization algorithm proposed and abundant traffic data mined, a scheme that combines genetic algorithms and traffic information with rule-based EMSs was developed, and the high fuel economy and low emission were realized [12,13,14]. However, it is still difficult to satisfy the fitness of conditions under them; hence, vehicular energy conservation potential needs to be developed.

Global optimization-based EMSs complete vehicular energy management under known working conditions. The intelligent traffic system (ITS) merging geographic information system (GIS), a global positioning system (GPS), enormous amounts of traffic flow data, and high-performance traffic modeling technology have developed rapidly, inspiring the proposal of the global optimization-based EMS [15]. Mainstream methods include dynamic programming (DP) [16], reinforcement learning (RL) [17], quadratic programming (QP) [18], and Pontryagin’s maximum principle (PMP) [19]. DP, considering the restriction of the Bellman equation, has found the global-optimization solution under separate questions [20]. On account of the simplification of the high non-linear problems, DP has been adopted widely by researchers. RL seeks the optimal solution based on the state probability function [21]. QP is suitable for situations where the cost functions can be described as quadratic functions, which look for optimal solutions according to the distribution of energy [22]. PMP (fully considering the bound) solves the best solution under the deterministic target function [23]. Although globally optimized EMSs can solve the optimal power distributions under random working conditions, the high requirements for time costs and calculation costs from the complexities of the automotive dynamic models constrain the online applications [24]. Consequently, the results of the globally optimized EMS are regarded as the performance benchmarks of other EMSs in general [25].

To overcome the limitations of the calculation costs from global optimization-based EMSs and the low efficiency brought about by rule-based EMSs, the instantaneous optimization-based EMS is proposed and developed. It adjusts the power distribution with the driving condition and component states during the per-control period to obtain the optimal energy consumption every time, which is mainly divided into EMSs based on the model predictive control (MPC) and equivalent consumption minimum strategy (ECMS). MPC uses optimization algorithms to solve the optimal control sequence in the predicted time domain based on the predicted future operating condition data and feeds the decision’s results back to the vehicle [26,27]. However, the MPC energy consumption optimization performance relies on a high precision reference speed or SOC with high computational costs, which limits its practical application. In contrast to MPC, the ECMS is widely used due to its low computational efforts and high real-time performance. It converts real-time electric consumption into equivalent instantaneous fuel consumption through the equivalent factor, solves the total fuel consumption composing the real-time equivalent fuel consumption and engine fuel consumption, and achieves real-time minimum total fuel consumption by controlling the engine and battery power allocation to obtain the optimal control decision [28,29,30]. The ECMS factor of ECMS usually needs to be calibrated for the representative working conditions. However, a single equivalent factor cannot satisfy the adaptive control of power components under different operating conditions, so adaptive ECMS is proposed to improve the working condition adaptabilities of vehicles.

The critical technologies of A-ECMS can be divided into two parts, adaptation optimization of equivalence factors and sensing for vehicle driving status. With the development of ITS and vehicle-road cooperation [31], large amounts of traffic sensor data are generated, making it possible to predict future traffic conditions based on historical traffic data [32,33,34]. Currently, the vehicle energy consumption optimization scheme integrates traffic information, specifically, the traffic data are utilized to predict the vehicle energy demand, vehicle speed conditions, or vehicle driving cycles, which guide the control actions of ECMS. Some scholars simply use the predicted vehicle states for ECMS adjustment, which obtains the vehicle’s demand power, speed, driving cycle, and SOC reference curve. Zeng et al. proposed an optimization-oriented adaptive equivalent consumption minimization strategy (A-ECMS) based on the demand power prediction realized by the iterative predictor. The proposed strategy periodically updates the optimal equivalent factor according to the predicted power through the local optimization process [35]. Liu et al. utilized the grey neural network to predict the future speed trend of the vehicle ahead. Based on the upper-level speed planning, the lower-level controller is responsible for the energy management of the vehicle based on the target speed, i.e., to generate the planned SOC reference track, and uses the fuzzy logic algorithm to update the equivalent factor in real-time according to the charge state difference [36]. Liu et al. adopted the nonlinear autoregression (NAR) algorithm of the moving horizon to predict short future driving cycles. The backpropagation (BP) neural network algorithm is used to identify the type of driving cycle, which provides a basis for adaptive ECMS [37]. Liu et al. utilized the adaptive network fuzzy inference system (ANFIS) model to train the SOC consumption curve under different driving cycles so that the vehicle could calculate the SOC reference curve in real-time according to the traffic data, and ensure that the A-ECMS strategy can adjust the equivalent factors according to the actual driving cycle. However, the vehicular driving state perception is mainly based on the vehicle’s state, i.e., for estimating the future driving conditions of the vehicle. However, such solutions ignore the influence of environmental information on the vehicle. More complex reference SOCs following control schemes also increase the computational loads of the onboard controller, so it is essential to enhance the vehicular perception of the driving environment and reduce the computational load of the onboard controller to enhance the real-time A-ECMS application capability.

To solve the above problems, this paper proposes an environment-perceived PHEV-based energy management strategy, EP-ECMS. EP-ECMS uses two-layer architecture to manage the energy system’s execution optimally. The high layer adopts an environmental perceiver based on a convolutional graph network and attention mechanism to identify the traffic state of the vehicle. The bottom layer utilizes an optimized ECMS to manage the energy system in the PHEV based on P2 configuration and to control the output of the electrical system and the fuel system; EP-ECMS calls the optimized equivalent factor according to the identified traffic state, and this operation is performed once more in a certain period of time to balance the performance and calculation costs, and fully exploit the energy-saving potential of PHEV.

The contributions of this paper are summarized as follows:

1. An advanced two-layer architecture of a PHEV energy management strategy (EP-ECMS) is proposed to realize the integration of traffic information and single-vehicle energy management, significantly improving the environmental adaptability of the vehicle and providing a new idea for single-vehicle energy management;
2. We developed a high-performance environmental perceiver based on a graph convolutional neural network and attention mechanism to accurately identify the traffic state, which guides the energy management of a single vehicle adapting to the environment;
3. The traffic conditions are classified by a self-organizing mapping neural network (SOM); the equivalent factors under various traffic conditions were optimized by the Harris hawk optimization algorithm (HHO) offline to improve the adaptability of EP-ECMS under different traffic states.

The rest of this paper is organized as follows. In Section 2, the vehicular configuration and component model are described. In Section 3, the structure of the proposed EP-ECMS is detailed. In Section 4, the control strategy simulation tests are performed to compare the regular EMS, the conventional ECMS, and the EP-ECMS. Section 5 draws the main conclusions of this study.

## 2. Model

### 2.1. Vehicle Configuration

**Figure 1:**
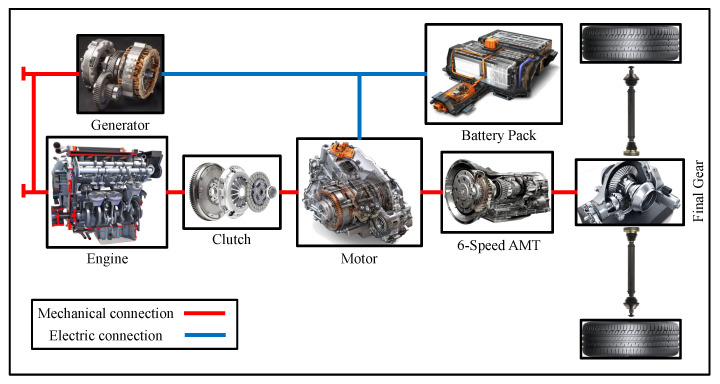
The architecture of the vehicle.

This paper is based on a parallel plug-in hybrid vehicle with the architecture shown in Figure 1. The vehicle’s fuel-related components consist of the engine, which is started by a high-speed generator. The electrical-related components consist of the electric motor and the battery. The engine and the electric motor are connected in parallel through a clutch. A six-speed mechanical automatic transmission transmits the driving force from the engine and the electric motor to the differential, which distributes it to the wheels. Table 1 shows the detailed parameters of the different components.

### 2.2. Mathematical Model of Vehicle Dynamics

The vehicle driving resistance equation and the driving demand power equation are calculated in Equations (1) and (2).

(1) $$F_{rest}=mgf\cos\alpha +mg\sin\alpha +\frac{C_{D}Av^{2}}{21.15}+\sigma m\frac{dv}{dt}$$

(2) $$P_{req}=\frac{v}{\eta_{d}}\left(\frac{mgf\cos\alpha }{3600}+\frac{mgf\sin\alpha }{3600}+\frac{C_{D}Av^{2}}{76140}+\frac{\sigma m}{3600}\frac{dv}{dt}\right)$$

where *m* is the overall vehicle mass, *g* is the gravitational acceleration, *f* is the rolling resistance coefficient, α is the slope, $C_{D}$ is the air resistance coefficient, *A* is the frontal wind area, *v* is the vehicle speed, σ is the rotating mass conversion coefficient, and $\eta_{d}$ represents the total efficiency of the drive system.

### 2.3. Mathematical Model of Fuel System

**Figure 2:**
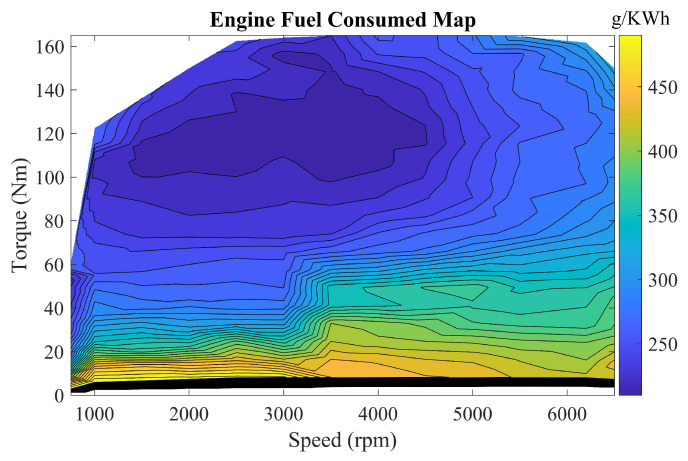
The fuel consumption map of the engine.

The engine, as a fuel-related component of the vehicle, provides traction power for the vehicle. The relationship between the non-linear dynamics of the engine, i.e., torque, speed, and fuel consumption can be obtained by checking the table, as shown in Figure 2, to obtain the engine operating point and fuel consumption.

The instantaneous fuel consumption of the engine is calculated as in Equations (3) and (4).

(3) $$\dot{m}{}_{f}=f_{eng}\left(T_{eng},\omega_{eng}\right)$$

where the mapping function $f_{eng}$ is obtained from the engine benchmark test, ${\dot{m}}_{f}$ denotes the fuel consumption rate (g/s), $T_{eng}$ is the engine torque, and $\omega_{eng}$ represents the engine angular velocity. In addition, the engine operating efficiency is described as

(4) $$\eta_{eng}\left(T_{eng},n_{eng}\right)=\frac{T_{eng}n_{eng}}{Q_{lhv}{\dot{m}}_{f}}$$

where $\eta_{eng}$ is the engine mechanical efficiency, $n_{eng}$ is the engine speed, and $Q_{lhv}$ is the low fuel heat value.

The engine transmits power to the wheels and drives the vehicle through the clutch, transmission, and main gearbox. The relationship between the torque and angular velocity at the engine and wheel end is expressed in Equations (5) and (6).

(5) $$T_{d\_eng}=T_{eng}i_{gb}\left(n\right)i_{fg}\eta_{t\_eng}$$

(6) $$\omega_{eng}=\omega_{wheel}i_{gb}\left(n\right)i_{fg}$$

where $T_{d\_eng}$ is the engine torque acting on the wheels, $T_{eng}$ is the torque provided by the engine in the fuel-related components, $i_{gb}$ and $i_{fg}$ represent the transmission ratio of transmission and main reducing gear, respectively, *n* is the transmission gear, $\eta_{t\_eng}$ is the engine-to-wheel transmission efficiency, $\omega_{eng}$ denotes the engine angular speed, and $\omega_{wheel}$ represents the wheel angular speed, respectively.

### 2.4. Mathematical Model of Electrical System

**Figure 3:**
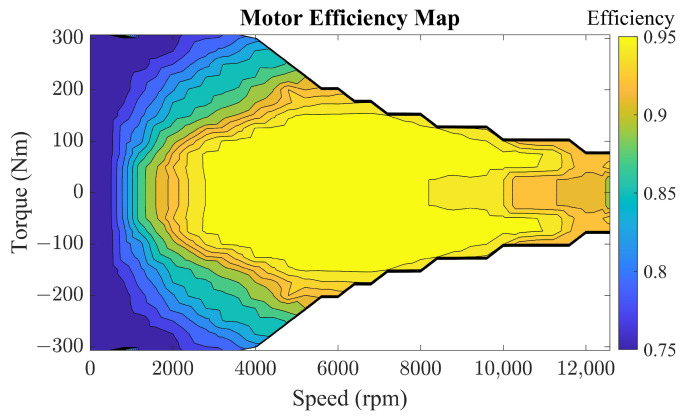
The motor efficiency map.

The motor as an electrical component provides traction power for the whole vehicle and its fast transient response is ignored. The relationship between the torque, speed, and efficiency is described by an efficiency diagram obtained from benchmark tests as shown in Figure 3. The efficiency of the motor is expressed in Equations (7)–(11).

(7) $$\eta_{em}=f_{em}\left(T_{em},n_{em}\right)$$

where the mapping function $f_{em}$ is obtained from the motor benchmark test, $T_{em}$ and $n_{em}$ represent the motor torque and speed, respectively. In the parallel PHEV, the motor can operate in the traction motor and generator states. In the traction motor mode, the relationship between the torque and power is expressed as follows.

(8) $$P_{em}=\frac{T_{em}\omega_{em}}{\eta_{mot}}$$

where $P_{em}$ is the motor power, $\omega_{em}$ is the motor angular speed, and $\eta_{mot}$ denotes the motor efficiency in traction mode. In the generator mode, the relationship between c torque and power is as follows:

(9) $$P_{em}=T_{em}\omega_{em}\eta_{gen}$$

where $\eta_{gen}$ indicates the motor efficiency in generator mode. The motor transmits power to the wheels and drives the vehicle through the transmission and the main reducer. The relationship between the torque and angular velocity at the motor and wheel end is expressed as.

(10) $$T_{d\_em}=T_{mot}i_{gb}\left(n\right)i_{fg}\eta_{t\_em}$$

(11) $$\omega_{mot}=\omega_{wheel}i_{gb}\left(n\right)i_{fg}$$

where $T_{d\_em}$ denotes the motor torque acting on the wheel, is the torque provided by the motor in the electrically related components, $T_{mot}$ represents the motor-to-wheel transmission efficiency, and $\omega_{mot}$ is the motor angular velocity.

In this paper, a lithium-ion battery is used, the effects of temperature and aging are ignored, and a simple equivalent circuit model is used to describe the battery performance. The output voltage of the battery is calculated in Equations (12) and (13).

(12) $$U=U_{oc}-I\cdot R_{int}$$

where *U* denotes the output voltage, $U_{OC}$ is the open circuit voltage, *I* is the battery current, and *R* represents the internal resistance of the battery. Then, the battery SOC is calculated as follows.

(13) $$SOC=-\frac{U_{oc}-\sqrt{U_{oc}-4R_{int}P_{batt}}}{2R_{int}Q_{batt}}$$

where $P_{batt}$ is the battery power and $Q_{batt}$ indicates the battery capacity (Ah). The value range of SOC is [0, 1]. When SOC = 0, the battery is fully discharged, and when SOC = 1, the battery is fully charged.

## 3. Implementation of Environmental Perceptive ECMS

**Figure 4:**
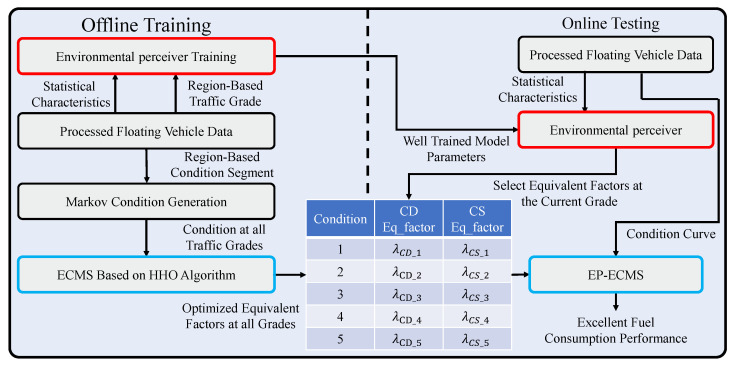
The architecture of EP-ECMS.

In this paper, for the complex nonlinear system composed of multiple power sources in P2 parallel PHEV, an energy management control system based on the perception of traffic state is proposed to combine traffic state information with energy management to fully utilize the energy-saving potential of PHEV in multiple environments. The architecture is shown in Figure 4. Considering that ECMS has an excellent balance between global optimization and instantaneous optimization, ECMS is selected as the core algorithm of energy management in this paper. In order to increase the adaptability of the environment and reduce the computational effort of a single vehicle, the regional traffic state obtained by the computing center deployed at the edge of the road was adopted to guide the energy management control of a single vehicle.

The offline design consists of three steps. The first step is to define the regional traffic state and train an environment-aware model. The environment-aware model is built based on a deep learning network, which can capture the spatiotemporal correlation of traffic data at the same time to achieve higher accuracy in traffic state perception. Then, the representative working conditions are generated for different grades of traffic data. The segments of the conditions in the area corresponding to all grades are counted, and the feature conditions of the corresponding grades are generated based on the classical Markov method. Finally, the equivalent factors in the ECMS strategy are trained offline by the HHO algorithm based on the feature conditions of different classes.

For the online test, the proposed energy management control strategy consists of two modules, the ECMS as the core control algorithm and the well-trained environmental perceiver. First, the environmental perceiver senses the traffic grades in all regions of the map at regular intervals and transmits the grade to a single vehicle. Then, the control of energy management of the vehicle is completed by invoking the well-optimized ECMS equivalent factor corresponding to the grades.

### 3.1. Environmental Perceiver

**Figure 5:**
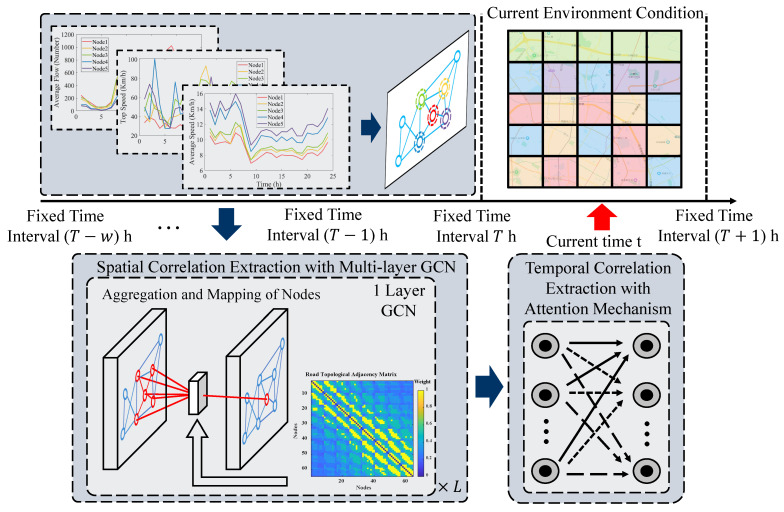
The architecture of the environmental perceiver.

The main task of environment sensing is to enable each vehicle to quantify the current traffic state, represented in the form of a hierarchy, which is shown in Figure 5. The perceived traffic grade guides the control of vehicle energy management. The road edge computing center completes the environmental sensing process through vehicle data collection, computation, and transmission. To reduce the complexity and computation when perceiving, we divide the city into regular sub-regions; vehicles in the same region are considered to be in the same traffic environment. Based on the defined area network, the environmental perceiver senses the grade of the traffic condition within each region in real-time. However, considering the traffic system’s scale and the network transmission’s delay, the environment sensing process is chosen to be performed at regular intervals.

#### 3.1.1. Pre-Processing of Traffic Data

Since the original data are the floating vehicle trajectory point data, pre-processing work is needed to serve the training task of the environmental perception model. The main pre-processing tasks include the statistics of area features and the generation of area classes. The area features are input to the perception model, and the area classes are used as prediction labels for the environmental perception model.

Features of the region are generated by counting the trajectory point data. Firstly, for the trajectory points $x_{i}=\left[t_{i},id_{v},id_{o},lat_{i},lng_{i}\right]\in R^{5}$, where $t_{i}$ denotes the time stamp, $id_{v}$ is the vehicle number, $id_{o}$ is the order number, $lat_{i}$ is the dimension, $lng_{i}$ is the longitude, the ratio of the distance and time difference from the previous trajectory point is calculated to obtain the speed $v_{i}^{t}$ at the corresponding moment. Second, in the spatial dimension, the city area is divided into N×N sub-regions. In the temporal dimension, the data are divided into different periods according to certain time intervals, and the time period boundaries are expressed as $T\in [1,T_{total}]$. The statistical characteristics of each area under all time periods are counted, and the statistical characteristics of the areas r∈[1,(N×N)] under time periods [T,T+1], specifically the average speed ${\bar{v}}_{r}^{T}$, the average flow $f_{r}^{T}$, and the maximum speed ${\hat{v}}_{r}^{T}$, are calculated in Equation (14).

(14) $$\left\{\begin{array}{c} {\bar{v}}_{r}^{T}=\frac{1}{m}\sum_{i=1}^{m}v_{i}^{t},\;\;\;t\in \left[T,T+1\right]\;\\ f_{r}^{T}=\frac{1}{m}\left(num_{T+1}-num_{T}\right)\;\\ {\hat{v}}_{r}^{T}=Top\left(v_{i}^{t}\right),\;\;\;t\in \left[T,T+1\right] \end{array}\right.$$

where *m* is the speed of all trajectory points in the time period [T,T+1], $num_{T}$ denotes the number of trajectory points in the region at the junction of the time period *T*, and Top(.) is the formula for taking the maximum value.

In order to objectively and comprehensively evaluate the traffic state, this paper adopts a self-organizing mapping neural network (SOM) to cluster the region’s features into a Class grades. Specifically, the three features of each region of the whole map at all moments are normalized to the maximum and minimum and then input into the SOM network to output the grade distribution of the whole map region at the corresponding moment. The calculation process is shown in the following Algorithms 1 and 2.
**Algorithm 1** Training process of self-organizing mapping neural network (SOM)**Input:** The normalized traffic condition sample *X*.**Output:** The well-trained SOM network.1:Initializing and normalize weight *W*, $W_{j},j=1,...,Class$; initial neighborhood radius $f_{neighbor}\left(1\right)=n_{0}=3$; initial learning rate $f_{learn}\left(1\right)=l_{0}=0.1$; initial max iterations MaxIter and iterations Iter.2:**while**Iter < MaxIter **do**3:   **for** *i* = 1 to T×N **do**4:     Choose the traffic condition sample $x_{i}\in X$.5:     **for** *j* = 1 to Class **do**6:        Calculate the Euclidean distance and select the nearest winning node.        $d_{ij}\left(x\right)=\sqrt{{\left(x_{i}-w_{j}\right)}^{2}}$7:        **for** *K* = 1 to $f_{neighbor}\left(1\right)$ **do**8:          Update the weight of the winning node and the neighbor nodes.          $w_{k}=w_{k}+f_{learn}\left(iter\right)\times f_{neighbor}\left(iter\right)\times \left(x_{i}-w_{k}\right)$9:          Update the number of iteration, learning rate, and neighborhood radius function.          $f_{neighbor}\left(Iter+1\right)=n_{0}\times \exp\left(-Iter/t_{1}\right),$          $t_{1}=MaxIter/\log\left(n_{0}\right)$          $f_{learn}\left(Iter+1\right)=l_{0}\times \exp\left(-Iter/t_{2}\right),$          $t_{2}=MaxIter$          Iter=Iter+110:        **end for**11:     **end for**12:   **end for**13:**end while**

**Algorithm 2** Testing process of self-organizing mapping neural network (SOM)
**Input:** The normalized traffic condition sample *X*; Trained SOM network.**Output:** The traffic grade *Y* corresponds to the traffic condition sample *X*.1:**for***i* = 1 to T×N**do**2:   Choose the traffic condition sample $x_{i}\in X$.3:   **for** *j* = 1 to Class **do**4:     Calculate the Euclidean distance and select the nearest winning node.     $d_{ij}\left(x\right)=\sqrt{{\left(x_{i}-w_{j}\right)}^{2}}$5:     The index j∗ of nearest node $w_{j*}$ is the class of sample $x_{i}$.6:   **end for**7:
**end for**



**Figure 6:**
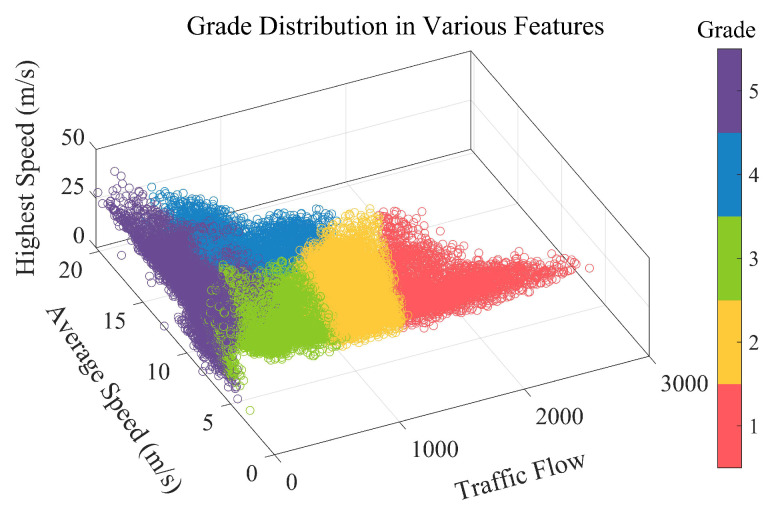
The distribution of five classes in the three dimensions of average speed, average flow, and maximum speed.

The distribution of classes is shown in Figure 6. The figure shows the distribution of five classes in the three dimensions of average speed, traffic flow, and maximum speed. Different classes show obvious boundaries in space, indicating that clustering results are effective. Different classes mainly reflect large differences in the two dimensions of average speed and traffic flow. First, based on the size of the traffic, the traffic is divided into class 1, class 3, class 4, and class 5 in order of distribution from small to large, corresponding to the traffic flow in the regions of [0, 500], [500, 1000], [1000, 1500], and [1500, 3000], and class 2 in the traffic. The distribution of class 2 in traffic covers the traffic ranges corresponding to classes 3 and 4, i.e., in the range [500, 1500]. Secondly, for the distribution of average speed, class 1 spans the widest range of [5, 20] km/h, class 2 is mainly distributed in the higher average speed part, which corresponds to the area [15, 20], and classes 3, 4, and 5 are distributed in the range of [5, 15] km/h with little difference in the average speed.

#### 3.1.2. Construction of Environmental Perception Model

The performance of environmental perception depends on the ability to capture spatial and temporal correlation characteristics in traffic data, and a graph convolutional neural network can capture complex non-Euclidean spatial correlation characteristics and is widely used in the field of traffic prediction; the attention mechanism benefits from the ability of global capture of time series and gradually becomes a research hotspot. Therefore, this paper tandemly connects the graph convolutional network and the attention mechanism to complete the construction of the environment perception model, and the model architecture is shown in Figure 6.

In this paper, we chose the GCN proposed by Kipf et al. [38] to capture spatially relevant features. Graph convolutional networks (GCNs) are widely used in traffic prediction tasks because they can effectively extract the correlation between nodes in the graph network space and are easy to compute. The graph convolutional neural network is based on the constructed graph network to perform aggregation and map each node input. For the first layer of GCN, the computational formula is shown in Equation (15).

(15) H(k)=ReLUD˜(k)−12W˜(k)D˜(k)−12H(k−1)θ(k)×LGCN

where ${\tilde{W}}^{\left(k\right)}=W^{\left(k\right)}+I$ is the adjacency matrix considering the self-loop, ${\tilde{D}}^{\left(k\right)}=\sum_{j}{\tilde{W}}_{ij}^{\left(k\right)}$ is the degree matrix, $\theta^{\left(k\right)}\in R^{d\times d^{\prime }}$ is a trainable parameter matrix, $W^{\left(k\right)}\in R^{n\times n}$ is the adjacency matrix of the graph network, $H^{\left(k-1\right)}\in R^{n\times d}$ is the node representation of the k−1th layer output, and $H^{\left(k\right)}\in R^{n\times d^{\prime }}$ is the node representation after feature extraction.

**Figure 7:**
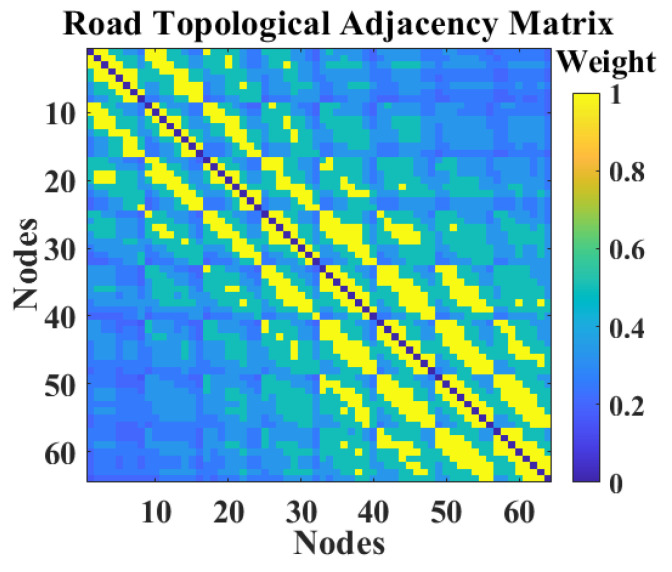
The road topological adjacency matrix.

From the formula, it is clear that the way the graph network is constructed affects the correlation weights between nodes. In this paper, the road network is constructed from the topological perspective of the road network space called the road network topological graph. It has been proved that this graph network can directly reflect the spatial distribution between roads and facilitate the extraction of spatial correlation characteristics. The road network topology diagram is represented as an undirected graph $G_{r}=\left(V,E,W_{r}\right)$, where the weight $\omega_{r}\left(i,j\right)\in \left(0,1\right]$ of the corresponding edge $e_{ij}$ is expressed as the reciprocal of the number of edges between road $v_{i}$ and road $v_{j}$. As the weight $\omega_{r}$ is closer to 1, the closer the two roads are to each other, and vice versa. The adjacency matrix $W_{r}$ composed of the weights of the $G_{r}$ is denoted in Equation (16), visualized as shown in Figure 7.

(16) $$W_{r}=\left[\begin{array}{cccc} 0 & \omega_{r}\left(1,2\right) & \cdots & \omega_{r}\left(1,N\right)\\ \omega_{r}\left(2,1\right) & 0 & \cdots & \omega_{r}\left(2,N\right)\\ \vdots & \vdots & \ddots & \vdots \\ \omega_{r}\left(N,1\right) & \omega_{r}\left(N,2\right) & \cdots & 0 \end{array}\right]$$

For the aspect of temporal correlation capture of traffic data, this paper uses the self-attention mechanism to complete the feature fusion of temporal dimension. The specific computational procedure is shown in Equation (17).

(17) $$Attention\left(X,X,X\right)=softmax\left(\frac{X\times X^{T}}{\sqrt{d_{k}}}\right)X$$

where *X* is the historical sequence, the attentional weights among the historical time points are obtained by calculating *X* and $X^{T}$ with the scaled dot product operation and softmax(.). Then the result is weighted and summed with *V* to obtain the road features with fused time correlation. The value of $d_{k}$ equals the feature dimension of *X*, which adjusts the result after multiplication to avoid gradient disappearance and improve computational efficiency.

After extracting temporal and spatial correlation, the road features must be mapped to the corresponding grade distribution of all nodes using a fully connected network. The calculation formula can be found in Equations (18)–(20).

(18) $$X_{out}={\left[linear\left(X_{in}\right)\right]}_{\times L_{Linear}}$$

(19) $$Linear^{\left(l\right)}\left(X_{in}\right)=ReLU\left(W^{\left(l\right)}\times X_{in}+b^{\left(l\right)}\right)$$

(20) $$Out=softmax(Reshape\left(X_{linear}^{out}\right))$$

where $X_{out}\in R^{\left(n\times Class\right)}$ is the feature vector of the mapping of the road to the traffic grade, linear(.) is the linear layer, and *L* is the number of stacked linear layers. Equation (19) represents the calculation process of the *l*th linear layer, where ReLU(.) is the ReLU activation function, $W^{\left(l\right)}\in R^{infeat^{\left(l\right)}\times outfeat^{\left(l\right)}}$ and $b^{\left(l\right)}\in R^{outfeat^{\left(l\right)}}$ denote the mapping weight and bias of the first layer, respectively. Finally, the output of the linear layer $X_{linear}^{out}\in R^{n\times Class}$ is operated with the operation of Reshape(.) and softmax(.) to obtain the probability distribution of all roads at different levels $Out\in R^{n\times Class}$, i.e., $Sum(Out_{i,:})=1,\;i=1,\cdots ,n$.

### 3.2. ECMS-Based Energy Consumption Control for PHEVs

The core control problem of PHEV is the distribution of the whole vehicle’s demand power to the engine and battery power. The core idea of ECMS is to calculate the equivalence factor, which converts the used electrical energy into equivalent fuel consumption, and to determine the optimal control variable, which determines the optimal engine to battery power distribution ratio for a given equivalence factor.

In the ECMS control process, the positive power (discharge) of the power battery means that energy is outputting, and the consumed energy needs to be replenished by the engine or the grid in the future, thus increasing the additional equivalent fuel consumption; the negative power (charge) of the power battery means that energy is being input, and the replenished energy is used to be released in the future, thus reducing some equivalent fuel consumption. The equivalent fuel consumption is expressed in Equations (21)–(23).

(21) $${\dot{m}}_{equ}\left(t\right)={\dot{m}}_{f}\left(t\right)+{\dot{m}}_{e2f}\left(t\right)$$

(22) $${\dot{m}}_{f}\left(t\right)=\frac{P_{eng}\left(t\right)}{\eta_{eng}\left(t\right)Q_{lhv}}=\frac{P_{req}\left(t\right)u\left(t\right)}{\eta_{eng}\left(t\right)Q_{lhv}}$$

(23) $${\dot{m}}_{e2f}\left(t\right)=\frac{s}{Q_{lhv}}P_{batt}\left(t\right)=\frac{s}{Q_{lhv}}P_{req}\left(t\right)\left(1-u\left(t\right)\right)$$

where ${\dot{m}}_{equ}$ is the instantaneous equivalent fuel consumption rate (g/s), ${\dot{m}}_{f}$ denotes the instantaneous fuel consumption rate (g/s), ${\dot{m}}_{e2f}$ is the equivalent fuel consumption rate for electrical energy conversion (g/s), s(t) is the equivalence factor, $P_{eng}$ denotes the engine power, $P_{req}$ is the overall vehicle demand power, and u(t) denotes the control variables.

In order to reduce the computational complexity, we simplify the unimportant factors in vehicle dynamics. In this paper, we only consider the effect of rolling resistance, air resistance and acceleration resistance power on the power demand of the whole vehicle, and Equation (2) can be simplified to Equation (24).

(24) $$P_{req}=P_{f}+P_{w}+P_{a}=\frac{1}{\eta_{t}}\left(\frac{mgfv}{1000}+\frac{C_{D}Av^{3}}{1632}+\frac{\sigma mv\alpha_{s}}{1000}\right)$$

where $P_{f}$ is the rolling resistance power, $P_{w}$ is the air resistance power, and $P_{a}$ is the acceleration resistance power.

Based on the proposed concept of equivalence factor, the energy management control objective of PHEV is formulated in Equation (25).

(25) $$\begin{array}{cc} \; & J\left(t\right)=min\int_{t_{0}}^{t_{e}}{\dot{m}}_{equ}\left(P_{req}\left(t\right),u\left(t\right),s,t\right)dt\\ \; & \;\;\;\;\;\;\;=min\int_{t_{0}}^{t_{e}}[{\dot{m}}_{f}\left(P_{req}\left(t\right),u\left(t\right),s,t\right)+{\dot{m}}_{e2f}\left(P_{req}\left(t\right),u\left(t\right),s,t\right)]dt \end{array}$$

In this paper, the minimum value of the objective function is solved by the Hamiltonian function as shown in the equation. The mathematical expressions of the constraints in the solution process are shown in Equations (26) and (27).

(26) $$H\left(P_{req}\left(t\right),u\left(t\right),s,t\right)={\dot{m}}_{f}\left(P_{req}\left(t\right),u\left(t\right),s,t\right)+{\dot{m}}_{e2f}\left(P_{req}\left(t\right),u\left(t\right),s,t\right)$$

(27) $$\begin{array}{c} H\left(P_{req}\left(t\right),u^{*}\left(t\right),s,t\right)\leq H\left(P_{req}\left(t\right),u^{*}\left(t\right),s,t\right)\;\\ s.t.\;\;u_{min}\leq u^{*}\left(t\right)\leq u_{max}\;\\ s_{min}\leq s\leq s_{max}\;\\ t_{0}\leq t\leq t_{e} \end{array}$$

where $u^{*}\left(t\right)$ is the optimal control sequence of vehicle demand power distribution ratio at time *t*, $u_{min}$ and $u_{max}$ denote the maximum and minimum bounds of power distribution ratio, respectively. $s_{min}$ and $s_{max}$ denote the minimum and maximum bounds of equivalence factor, respectively.

The optimal sequence of control variables $u^{*}\left(t\right)$ is solved in a finite set of Hamiltonian equations to obtain the minimum value of the Hamiltonian equation. The constraints of the actuating components, the constraints of the regenerative energy storage system and the constraints of the battery SOC need to be considered in the calculation process, and the calculation process and the main constraints are shown in Equation (28).

(28) $$\begin{array}{c} u^{*}\left(t\right)=argminH\left(P_{req}\left(t\right),u\left(t\right),s,t\right)\;\\ s.t.\;\;P_{batt\;\;\_\;\;min}\leq P_{batt}\left(t\right)\leq P_{batt\;\;\_\;\;max}\;\\ P_{eng\;\;\_\;\;min}\leq P_{eng}\left(t\right)\leq P_{eng\;\;\_\;\;max}\;\\ T_{eng\;\;\_\;\;min}\leq T_{eng}\left(t\right)\leq T_{eng\;\;\_\;\;max}\;\\ T_{mot\;\;\_\;\;min}\leq T_{mot}\left(t\right)\leq T_{mot\;\;\_\;\;max}\;\\ \omega_{eng\;\;\_\;\;min}\leq \omega_{eng}\left(t\right)\leq \omega_{eng\;\;\_\;\;max}\;\\ \omega_{mot\;\;\_\;\;min}\leq \omega_{mot}\left(t\right)\leq \omega_{mot\;\;\_\;\;max}\;\\ SOC_{min}\leq SOC\left(t\right)\leq SOC_{max}\;\\ u_{min}\leq u\left(t\right)\leq u_{max}\;\\ t_{0}\leq t\leq t_{e} \end{array}$$

where $P_{batt\;\;\_\;\;min}$ and $P_{batt\;\;\_\;\;max}$ denote the minimum and maximum power boundaries of the battery in turn, and the engine power $P_{eng}$, engine torque $T_{eng}$, motor torque $T_{mot}$, engine speed $\omega_{eng}$ and motor speed $\omega_{mot}$ constraints depend on the torque and speed limits of the engine and motor. $SOC_{\min}$ and $SOC_{\max}$ denote the minimum and maximum values of the SOC of battery, respectively.

Based on equivalence factors, current researchers have focused more on adjusting equivalence factors by individual vehicle information (driving state and component state), such as assigning equivalence factors based on identified road conditions [39] and adjusting equivalence factors based on online prediction information [40]. The accuracy of road condition identification and vehicle speed estimation affects the environmental adaptability of the energy management strategy and places increasing demands on the computational power of the vehicle controller as the control algorithm becomes more complex. In the next section, we propose EP-ECMS to capture macroscopic traffic information and guide a single vehicle for energy management control by formulating a more efficient and accurate environmental perceiver.

### 3.3. The Optimization Process of EP-ECMS

Many hardware facilities are deployed with the development of intelligent transportation systems (ITS). ITS integrates information, data communication, and sensing technology to realize the close cooperation between workshop communication and the vehicle environment. In this paper, the prediction of the raffic state levels is accomplished by the environment-aware model proposed by Section 3.1, and the corresponding optimized ECMS equivalent factors are selected based on the predicted levels. In the offline process, these equivalent factors are optimized by the swarm intelligence optimization algorithm of HHO. Therefore, the EP-ECMS architecture and the optimization algorithm are presented in the following sections.

#### 3.3.1. The Operation Process of EP-ECMS

**Figure 8:**
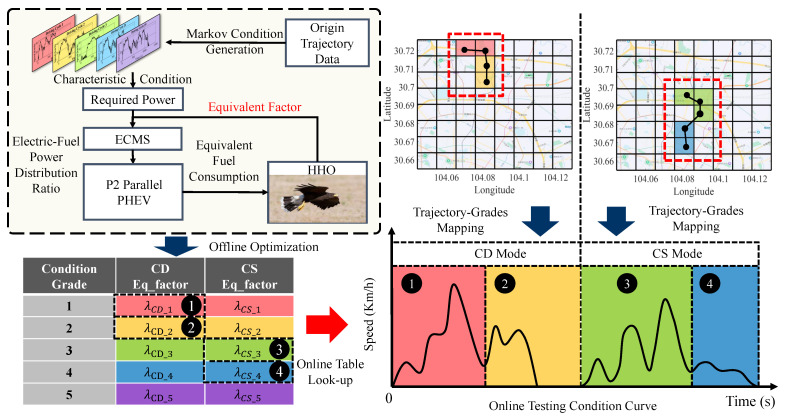
The operation process of EP-ECMS. Especially, The grid diagram on the upper right shows an example of the vehicle path. Among them, the red box represents the driving track of the vehicle. The vehicle is in CD mode when driving in the front section, as shown in the grid diagram on the left, while the vehicle is in CS mode in the rear section, as shown in the grid diagram on the right.

In the EP-ECMS, the call of equivalence factors is based on the current traffic grade of the vehicle identified by the environmental perceiver, and the operation process is shown in Figure 8. Firstly, after collecting the working condition fragments under different classes, the Markov chain method [41] is used to generate the characteristic working conditions of the corresponding class; then, based on the generated characteristic working conditions, the HHO algorithm is adopted to complete the optimization of the equivalent factors of the corresponding working conditions, as shown in Section 3.3.2; finally, the actual working condition test, according to the current vehicle class, is identified by the environment perception. Finally, during the actual test, the offline optimization table is called online according to the current vehicle level identified by the environment sensor, so as to control the energy consumption performance of the whole vehicle with a high environment-adapted energy allocation strategy.

#### 3.3.2. HHO-Based Optimization of ECMS Equivalence Factors

By analyzing the process of the Harris hawk predation, Heidari et al., in 2019, proposed HHO as a population intelligence optimization algorithm [42]. HHO has a strong global search capability and requires fewer parameters to be adjusted. In this paper, the equivalence coefficients are optimized for the characteristic working conditions under different classes. The values of the two equivalence factors for CD and CS modes are optimized according to the different operating modes of PHEVs of P2 configuration. In the HHO algorithm, the position of the Harris hawk represents the equivalence factor in the optimization process, and the equivalent fuel consumption is represented by the adaptation of the prey position, so the smaller the equivalent fuel consumption is, the lower the adaptation is.

The Harris eagle optimization algorithm consists of three main components: the search phase, the transition between search and exploitation, and the exploitation phase. In the exploration phase, all individuals in the hawk flock are in a waiting state, and by examining and monitoring the search space in order to find prey, different perching strategies are selected based on random values, as modeled in Equation (29).

(29) $$X\left(t+1\right)=\left\{\begin{array}{c} X_{rand}\left(t\right)-r_{1}\left|X_{rand}\left(t\right)-2r_{2}X\left(t\right)\right|,q\geq 0.5\;\\ |X_{rabbit}\left(t\right)-X_{m}\left(t\right)|-r_{3}\left[lb+r_{4}\left(ub-lb\right)\right],q<0.5 \end{array}\right.$$

where X(t) and X(t+1) denote the positions of individuals at the current and next iteration, respectively, *t* is the number of iterations, $X_{rand}\left(t\right)$ is the randomly selected position, and $X_{rabbit}\left(t\right)$ is the prey position, i.e., the positions of individuals with optimal fitness. $r_{1}$, $r_{2}$, $r_{3}$, $r_{4}$, and *q* are random numbers between [0, 1]. *q* is used to select the adopted strategy, $X_{m}\left(t\right)$ is the average position of the individuals, and the expression is shown in Equation (30).

(30) $$X_{m}\left(t\right)=\sum_{k=1}^{M}X_{k}\left(t\right)/M$$

where $X_{k}\left(t\right)$ is the position of the *k*th individual in the population and *M* is the population size.

The energy based on prey escape varied between exploitation behaviors during the search and exploitation transition phase. The energy of prey escape was significantly reduced during the escape behavior. The escape energy is defined in Equation (31).

(31) $$E=2E_{0}\left(1-\frac{t}{T}\right)$$

where $E_{0}$ is the initial energy of the prey, which is a random number between [−1, 1] and is updated automatically at each iteration. *t* is the number of iterations, and *T* is the maximum number of iterations. When |E|≥1 enters the search phase, |E|≤1 enters the development phase.

During the development phase, the Harris Hawk began to make surprise attacks on its prey. Based on the energy and behavior of the prey’s escape, the Harris hawk evolved four attack strategies. The prey tried to escape from the danger during the chase by indicating the chance of the prey escaping before the raid as *r*; r≥0.5 indicates a successful escape and vice versa. Specifically, the four strategies are shown below.

Soft siege: The prey still has enough energy and checks to escape from the chase by some random misleading jumps, i.e., r≥0.5 and |E|≥0.5. In this case, Harris’s Hawk uses the soft siege strategy to exhaust the rabbit and then makes a surprise attack, see Equation (32).

(32) $$X\left(t+1\right)=X_{rabbit}\left(t\right)-X\left(t\right)-E\left|J\cdot X_{rabbit}\left(t\right)-X\left(t\right)\right|$$

where *J* is the prey escape process jump distance, J=2×(1−rand).

Hard siege: The prey is very tired and the escape energy is low. The prey does not have enough energy to rid itself of the pursuit, and there is no chance of escape, i.e., r≥0.5 and |E|<0.5. At this time, Harris hawk uses the hard siege method of hunting, the formula is in Equation (33).

(33) $$X\left(t+1\right)=X_{rabbit}\left(t\right)-E\left|X_{rabbit}\left(t\right)-X\left(t\right)\right|$$

Soft siege with a progressive fast dive: The prey has a chance to escape and has sufficient escape energy. For this situation, Harris’s hawk needs to form a gentle siege with a progressive fast dive before attacking, at this time r<0.5 and |E|≥0.5. Specifically, two strategies were adopted based on prey adaptations, summarized in Equation (34).

(34) $$X\left(t+1\right)=\left\{\begin{array}{c} Y\;\;\;\;if\;\;\;\;fitness(Y)<fitness(X(t))\\ Z\;\;\;\;if\;\;\;\;fitness(Z)<fitness(X(t)) \end{array}\right.$$

where *Y* and *Z* correspond to two strategies, respectively, which are shown in Equation (35).

(35) $$Y=X_{rabbit}\left(t\right)-E\left|J\cdot X_{rabbit}-X\left(t\right)\right|$$

If the adaptation does not improve after this measure is implemented, an alternative strategy is implemented.

(36) Z=Y+S×LF(D)

where *D* is the spatial dimension and *S* is a random vector of 1×D, i.e., S=randn(1,D), LF(D) is the Levy flight function, which is shown in Equation (37).

(37) $$LF\left(x\right)=0.01\times \frac{u\times \sigma }{|v{|}^{\frac{1}{\beta }}},\sigma ={\left(\frac{\Gamma \left(1+\beta \right)\times \sin\left(\frac{\pi \beta }{2}\right)}{\Gamma \left(\frac{1+\beta }{2}\right)\times \beta \times 2^{\frac{\beta -1}{2}}}\right)}^{\frac{1}{\beta }}$$

where *u* and *v* are random numbers uniformly distributed within [0, 1], β=1.5.

Tough encirclement with progressive fast dive: When r<0.5 and |E|<0.5, the prey has a chance to escape, but the escape energy is insufficient, so the Harris hawk forms a tough encirclement before the raid, and gradually reduces the average distance to the prey on the basis of stabilizing the field. The equations are shown in Equations (38)–(40).

(38) $$X\left(t+1\right)=\left\{\begin{array}{c} Y\;\;\;\;if\;\;\;\;fitness(Y)<fitness(X(t))\\ Z\;\;\;\;if\;\;\;\;fitness(Z)<fitness(X(t)) \end{array}\right.$$

(39) $$Y=X_{rabbit}\left(t\right)-E\left|J\cdot X_{rabbit}-X\left(t\right)\right|$$

(40) Z=Y+S×LF(D)

## 4. Simulation and Evaluation

In this section, the environmental sensing and energy management strategies are tested separately, where the high accuracy of environmental sensing provides the basis for the vehicle to control the energy distribution with a rational strategy, and the optimization of the energy management strategy is the core of the energy management by the vehicle. Therefore, it is necessary to test both.

### 4.1. Environmental Perception Model Testing

The highly accurate environmental perceiver depends on the architecture and parameters of the perception model. Based on the set parameters, we tested the performance of the environmental perception, which is described below in terms of both the model configuration and the testing of the environmental perception.

#### 4.1.1. Model Configuration

This experiment is conducted based on the floating vehicle data from the big data platform of DiDi, and the vehicle trajectory data of the zone from 1 November to 30 November 2016 were selected as the source data. The average vehicle speed, average traffic flow, maximum vehicle speed, relative average traffic flow, and relative average vehicle speed of the area were counted once an hour according to the divided sub-regions, and the total data set size was 720 groups.

The hyperparameters and training parameters of the environmental perception model are shown in Table 2.

#### 4.1.2. Performance of Environmental Perceiver

In this paper, the quadratic weighted kappa coefficient (Kappa coefficient) was used as the evaluation index of the prediction effect, where the quadratic weighted kappa coefficient indicates the consistency of the predicted grade with the true grade distribution, and this coefficient characterizes the accuracy and deviation of the prediction. The calculation process is based on the confusion matrix, which takes values between −1 and 1. The closer the value is to 1, the higher the consistency of the prediction grade results. The accuracy and weighted kappa coefficients are calculated as shown in Equations (41)–(43).

(41) $$ACC=\frac{1}{n}\sum_{t=1}^{n}\left(1,\;\;if\;v_{t}={\tilde{v}}_{t}\;else\;0\right)$$

(42) $$KAPPA=\frac{P_{o}-P_{e}}{1-P_{e}}$$

(43) $$\left\{\begin{array}{c} P_{o}=\sum_{i=1}^{Class}\sum_{j=1}^{Class}\omega_{i,j}p_{i,j}\;\\ P_{e}=\sum_{i=1}^{Class}\sum_{j=1}^{Class}\omega_{i,j}p_{i,:}p_{:,j}\;\\ \omega_{i,j}=1-{\left(\frac{i-j}{Class-1}\right)}^{2} \end{array}\right.$$

Based on the test set data, this paper demonstrates the prediction performance of the environment perception, where the accuracy and kappa coefficient results are shown in Table 3. Finally, the prediction accuracy is 90.332%, and the kappa coefficient is 0.9511, which indicates that the environment perception shows a better prediction performance on different traffic levels, which provides a stable basis for the subsequent selection of energy management strategies.

**Figure 9:**
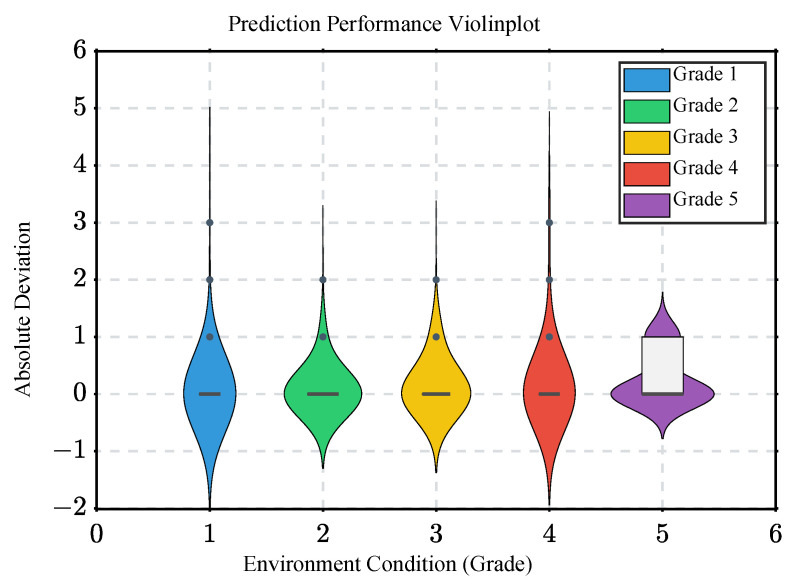
The prediction performance.

To judge the prediction performance under different grades, Figure 9 shows the state of the absolute difference distribution between the predicted grade and the true grade for all instances under different grades, presented in the form of a violin plot. It can be seen that the predictions for the traffic grades 1 to 4 are significantly better than that of grade 5, as reflected by the more concentrated quartiles. This situation is understood to be due to the fact that there are fewer traffic data for grade 5, which prevents the environment perception model from fully learning the corresponding features, thus causing a certain degree of misjudgment. Under the prediction task of grades 1 to 4, grades 2 and 3 are slightly better than grades 1 and 4, as reflected by a greater concentration on the grade difference of 0, along with the presence of fewer outliers.

**Figure 10:**
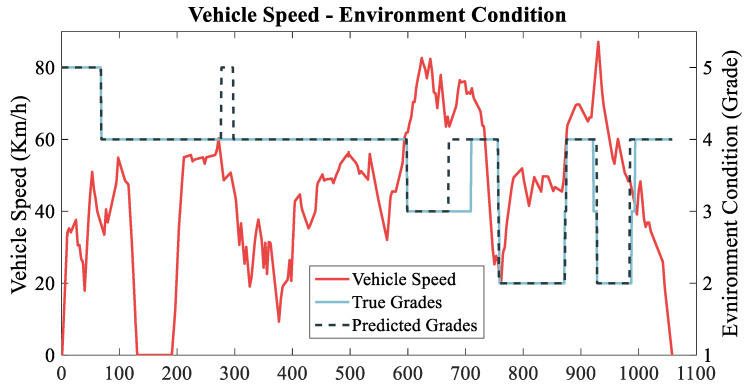
The prediction effect display.

In order to show the prediction of traffic status level more intuitively, Figure 10 shows the speed curve, original traffic grade curve, and predicted traffic grade curve corresponding to a randomly selected trajectory. From the figure, it can be seen that there are certain deviations and anomalies in the predicted grade distribution over time compared with the real grade, with the deviations basically differing by one grade in general and the anomalies lasting for a shorter period of time due to environmental disturbances more often. In general, there is some inaccuracy in prediction, but it does not affect the overall accuracy of the prediction and the consistency of the prediction results.

### 4.2. The Simulation Testing of Working Conditions

In order to verify the potential of EP-ECMS in terms of fuel economy, simulations were performed in Matlab/Simulink. In addition to EP-ECMS, this paper also compares a rule-based control strategy (RB) with a fixed equivalence factor ECMS. Four modes of operation are available for the charge-depleting (CD) and charge-sustaining (CS) stages: pure electric drive mode, engine drive mode, hybrid drive mode, and charging mode. In the pure electric drive mode, the electric motor is the only power source for the vehicle; in the engine drive mode, the engine drives the vehicle alone; the hybrid drive mode corresponds to the dual power source of the engine and the battery; and in the charging mode, the engine drives the vehicle and charges the battery at the same time.

In the constructed PHEV model, the initial battery SOC is 0.32 and enters the CS phase when the SOC is lower than 0.28, and the final battery SOC is controlled between 0.28 and 0.304.

In this paper, the algorithm’s adaptability to the environment and the optimization of energy consumption are tested by two sets of randomly selected actual trajectory data, respectively. In order to exclude the interference caused by the environment perception, the table look-up of the equivalence factor is completed by inputting the real traffic condition grade, and the corresponding lines are simulated by running in MATLAB/Simulink.

#### 4.2.1. Energy Optimization Assessment

In order to evaluate the performance of EP-ECMS for fuel economy optimization, this paper compares the rule-based control strategy, the fixed equivalence factor ECMS control strategy, and the proposed EP-ECMS control strategy. We perform pre-processing work on a randomly selected floating vehicle trajectory, resulting in a speed profile of the 1040s. After the fuel consumption simulation test, the table shows the total fuel consumption (equivalent fuel consumption) for different energy management strategies. Compared with the conventional RB and fixed equivalent factor ECMS, EP-ECMS brings 25.18% and 5.03% savings in the vehicle’s equivalent fuel consumption, respectively.

**Figure 11:**
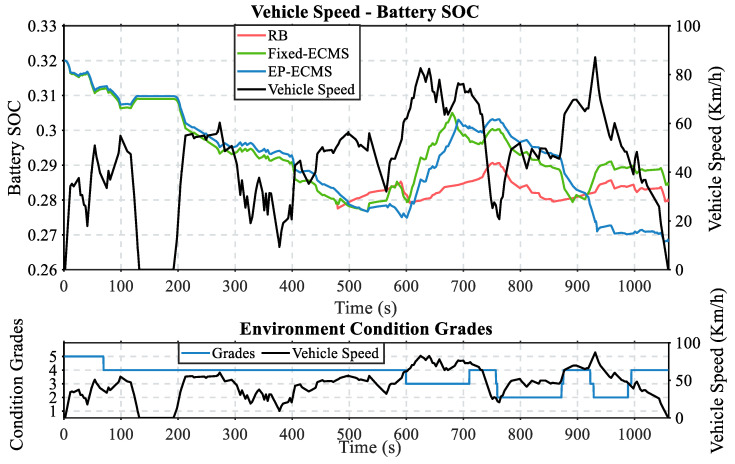
The first random working condition test result.

Figure 11 shows the driving speed versus SOC variation curves for a given trajectory route. The figure clearly shows that the SOC curves exhibited by the different methods have significant differences. First, comparing the rule-based control strategy with the ECMS-based control strategy, the SOC variation trend of the rule-based method is the flattest in the interval from 500 s to 1060 s, which is inappropriate in working conditions with more intense speed changes and reflects poor working condition adaptability. For example, in the 600 to 750 s range, the vehicle is in the high-speed range, and the SOC is low. The engine should be responsible for driving the vehicle and charging the battery. The charging power of the RB method is too low to supplement the SOC to a reasonable range. Second, comparing the result proves that EP-ECMS has higher environmental adaptability than the classical ECMS control strategy. Specifically, for the 200–300 s range, the vehicle is in the high-speed range. EP-ECMS uses the engine more to drive the vehicle and thus consumes less battery power; for the 540–600 s range, the vehicle is in the low-speed range. EP-ECMS uses the motor more to drive the vehicle and thus avoids the engine being in the inefficient range; for the 900–1060 s range, the vehicle speed is in the low-speed range, EP-ECMS uses the motor more to drive the vehicle and thus avoids the engine being in the inefficient range. For the 900–1060 s range, the vehicle speed is in the low-speed range. EP-ECMS reduces engine inefficiency by controlling the electric motor as the main driving force of the vehicle.

#### 4.2.2. Assessment of the Working Condition of Power Components

**Figure 12:**
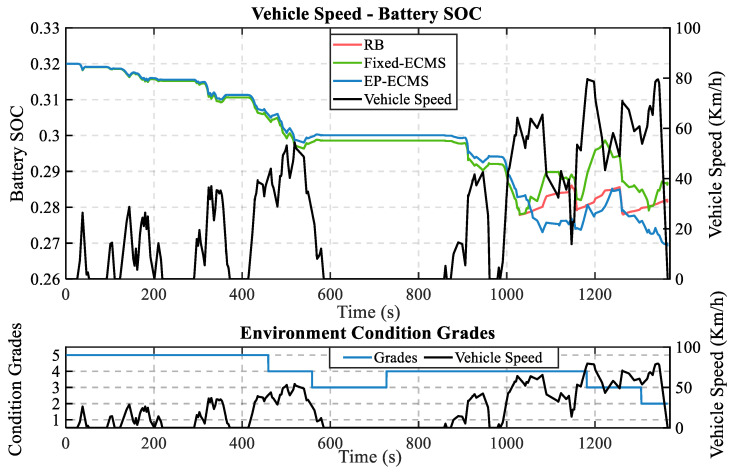
The second random working condition test result.

To further analyze the control effect of energy management strategy on the working point of power components, more trajectory data were randomly selected and pre-processed to obtain a speed curve of length the 1370s, and an energy consumption simulation test was conducted based on this working condition, which is shown in Figure 12. The trajectory is shown in the figure. Specifically, EP-ECMS improves by 25.73% compared to RB and 7.25% compared to ECMS, which again shows that EP-ECMS can effectively improve the fuel economy of the whole vehicle.

**Figure 13:**
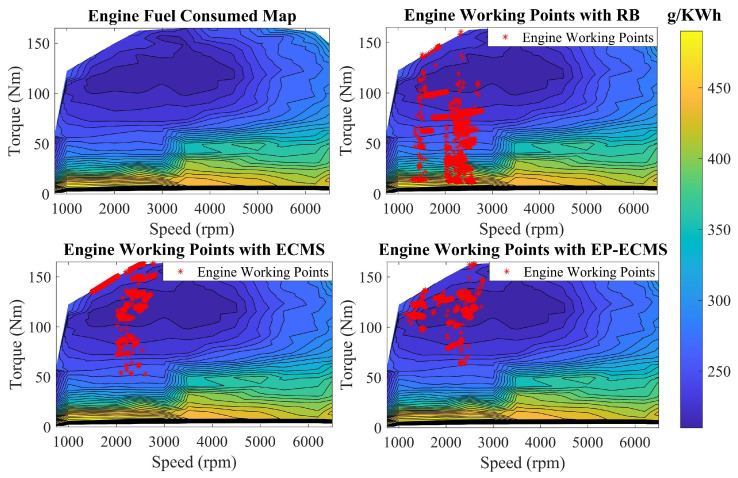
The engine working point in the efficiency map.

Figure 13 shows the distribution of engine operating points in different energy strategies. Comparing the rule-based energy management strategy with the classical ECMS, the engine operating points of the proposed EP-ECMS are more concentrated in the low fuel consumption rate part. Under the rule-based energy management strategy, the torque and speed of the engine are more distributed in the low-value range, indicating that the engine drives the vehicle more in low-speed operating conditions, and the engine cannot give full play to its performance in the high-speed operating conditions. The fixed equivalence factor ECMS is more distributed in the high-efficiency interval compared with the rule-based method. However, it has some operating points distributed on the outer characteristic curve, which affects the overall operating point efficiency. In addition, the engine efficiency worsens due to some low-torque operating points of the engine. For EP-ECMS, the controlled engine operating points are more concentrated in the high-efficiency part than the fixed-factor ECMS, specifically, less distributed in the outer characteristic curve and the low-torque part.

**Figure 14:**
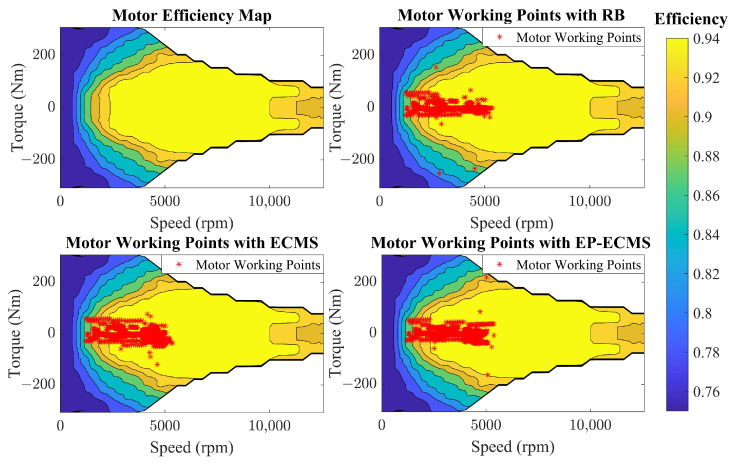
The motor working point in the efficiency map.

Figure 14 shows the distribution of motor operating points for different energy management strategies. The integrated distribution of efficiency intervals over torque reveals that EP-ECMS has a stronger balance. For the efficiency interval distribution, the work of EP-ECMS and ECMS based on fixed equivalence factor is distributed in the high-efficiency interval. At the same time, the rule-based control strategy has some points with an efficiency below 80%. For the distribution of torque intervals, the distribution of EP-ECMS is more concentrated in the low-torque area than that of fixed-equivalent factor ECMS above 2500 rpm, which means that the electric motor is more involved in the vehicle driver as an auxiliary to ensure the engine works in the appropriate range in the corresponding high-speed range.

The above paper compares the simulation test results of EP-ECMS with the baseline from multiple perspectives, which are the SOC optimization curve, engine operating point, and motor operating point distribution, respectively. The results show that EP-ECMS is very effective at optimizing the energy consumption control of PHEVs. PHEVs can adjust the equivalence factor according to the perceived traffic conditions, thus showing excellent power component operating point distributions.

## 5. Conclusions

In this paper, an energy management control architecture based on environmental perception is proposed to optimize the management of the P2 parallel PHEV energy system by combining the working condition categories identified by the environmental perception with ECMS to improve the environmental adaptability of PHEV and, thus, fully utilize the energy-saving potential. The overall control is divided into offline optimization and online testing. The offline process includes training the environment perception based on GCN and attention mechanism and optimizing the equivalence factors under different environmental grades. In the online test, the table look-up of the equivalence factor is completed by the environment level identified by the environment perception, and the energy management control is completed based on the optimized equivalence factor. This paper compares the simulation results using MATLAB/Simulink, and the improvement is 7.25% compared to the conventional ECMS economic model.

However, the current state of the formulated environment does not accurately reflect the real environment the vehicle is in, and the potential of the control strategy needs to be further developed. In future research, more accurate perception criteria for the single-vehicle environment should be considered to improve the economy of PHEVs further.

## Figures and Tables

**Table 1:** Vehicle configuration parameters.

Component	Parameter	Value	Component	Parameter	Value
Engine	Displacement	2.0 (L)		Capacity	21 (Ah)
	Type	In-line four-cylinder gasoline engine	Battery	Type	Lithium
	Maximum power	103 (kW) @6200 (r/min)	Generator (starter)	Nominal voltage	300 (V)
	Maximum torque	160 (N m) @2500 6000(r/min)		Maximum power	8.5 (kW)
Motor	Maximum power	124 (kW)		Maximum torque	45 (N m)
	Maximum torque	305 (N m)		Maximum speed	5000 (r/min)
	Maximum speed	12,480 (r/min)	Gearbox	Type	6 Speed AMT

**Table 2:** Hyperparameters and training parameters of the environmental perceiver.

Detailed Parameter Setting			
Number of roads	64	Optimizer	ADAM
features of roads	3	Learning rate	$5\times 10^{-4}$
Historical length	24	Weight decay	$1\times 10^{-3}$
GCN layer $L_{GCN}$	2	Batch size	24
GCN hidden dimension	32	Training Epoch	500
Linear hidden dimension	64	Training set size	576
Linear layer $L_{linear}$	2	Validation set size	72
Number of traffic condition grades	5	Testing set size	72

**Table 3:** The performance of the environmental perceiver.

Perception Performance	Value
Accuracy	90.332%
Quadratic Weighted Kappa Confidence	0.9511

## Data Availability

Not applicable.

## Abbreviations

The following abbreviations are used in this manuscript:

GHG	greenhouse gas
HEV	hybrid electric vehicle
PHEV	plug-in hybrid electric vehicle
EMS	management strategy
ECMS	equivalent consumption minimum strategy
MPC	model predictive control
A-ECMS	adaptive-equivalent consumption minimum strategy
SOC	state of charge
ITS	intelligent traffic system
GIS	geographic information system
GPS	global positioning system
DP	dynamic programming
QP	quadratic programming
PMP	Pontryagin’s maximum principle
RL	reinforcement learning
NAR	nonlinear autoregression
BP	backpropagation
ANFIS	adaptive network fuzzy inference system
GCN(s)	graph convolutional network(s)
SOM	self-organizing mapping neural network
HHO	Harris hawk optimization
RB	rule-based
CD	charge depleting
CS	charge sustaining
